# Comparison of the anatomical and functional success of fascia and perichondrium grafts in transcanal endoscopic type 1 tympanoplasty

**DOI:** 10.1186/s40463-019-0386-z

**Published:** 2019-11-27

**Authors:** Kadir Özdamar, Alper Sen

**Affiliations:** 1 Department of Otorhinolaryngology - Head and Neck Surgery, Private Lotus Hospital, Şanlıurfa, Turkey; 2 Department of Otorhinolaryngology - Head and Neck Surgery, Medical Faculty, Harran University, Şanlıurfa, Turkey

**Keywords:** Endoscopic tympanoplasty, Perichondrium, Fascia, Transcanal

## Abstract

**Background:**

There are no studies in the literature, comparing the functional and anatomical successes of the use of fascial and perichondrial grafts in endoscopic type 1 tympanoplasties.

**Objectives:**

To compare the anatomical and functional outcomes of grafting with the fascia of the temporalis muscle and with the perichondrium of the tragal cartilage in patients undergoing primary transcanal type 1 tympanoplasty with endoscopy.

**Methods:**

We enrolled a total of 151 patients (80 females and 71 males with a mean age of 26.0 ± 9.3 years in the age range between 18-57) with MERI scores ranging from 1 to 3 and who underwent a transcanal endoscopic type 1 tympanoplasty without tympanomeatal flap elevation. The patients were assigned to two groups according to the type of the graft used. The patients were assigned to either the tragal cartilage perichondrium group (Group A) or the fascia of the temporal muscle (Group B). The groups were compared according to the pre- and postoperative air-bone gaps and to the status of the tympanic membrane.

**Results:**

There were no statistically significant differences in the distribution of the age, gender, localization, MERI scores, the duration of the operation, and the size of the perforation (all *p* values> 0.05). The pre-operative air-bone gap values of Group A and B did not show a statistically significant difference (*p* = 0.073). The postoperative improvement in the air-bone gap value did not demonstrate a significant difference between Group A and B (*p* = 0.202). The graft retention rates were 94.9 and 97.2% in Group A and in Group B respectively. There were no statistically significant differences between the two groups in terms of the graft retention success rates (*p* = 0.743).

**Conclusion:**

Perichondrium and fascia were suitable for use in endoscopic tympanoplasties.

## Introduction

The tympanic membrane bears characteristic anatomical and physical features. The acoustic properties of the tympanic membrane have direct effects on the sound transmission mechanisms of the ossicular chain. Tympanoplasty is a surgical procedure to reconstruct the tympanic membrane after perforations and to correct hearing losses [1, 2]. In the literature, the first surgical intervention to treat a tympanic membrane perforation was reported to be performed by Berthold in 1878 [3] and the first predecessors of today’s modern tympanoplasty techniques were developed by Zöllner [4] and Wullstein [5].

There are a number of studies on tympanoplasties in the literature, introducing novel techniques and methods. Today, varying types of grafts are available for use in tympanoplasties. The most frequently used ones are biological materials and they include the fascia of the temporal muscle, tragal cartilage, perichondrium, fat, skin, and veins [1, 2]. The types of the grafts to be used vary depending on the experiences and preferences of the surgeons working at different institutions [6, 7]. The fascia of the temporal muscle is the most commonly used graft in primary tympanoplasties with success rates of 6 8-97% for graft retentions [8, 9].

The modern tympanoplasty techniques were defined in the 1950s with the introduction of operating microscopes [10]. The majority of the cases may require a retroauricular incision and tissue resection in microscopic tympanoplasties. The microscopic tympanoplasty technique is commonly used today most frequently to reconstruct large tympanic membrane perforations located anteriorly. However, a retroauricular scar formation, displacement of the pinna anteriorly, and the development of significant pain in the patients are the disadvantages of the microscopic tympanoplasties [10–12]. Currently, the minimally invasive techniques have gained popularity. There is an increasing interest in the minimally invasive techniques in otologic and neuro-otologic surgeries. Endoscopic ear surgeries were introduced in the 1990s and they gained popularity in otology. Endoscopically, it is possible to elevate a tympanomeatal flap via the transcanal route, however, it is also possible to perform a tympanoplasty without the elevation of the tympanomeatal flap [13]. The anatomical structures of the middle ear, the anterior and posterior epitympanic spaces, tympanic sinus, and the facial recess can be visualized more clearly by endoscopy [13, 14].

There are a number of studies available in the literature comparing the functional and anatomical outcomes after grafting by microscopic tympanoplasties [10–12, 15]. While a functional success is accepted to achieve an improvement in hearing along with a reduction in the air-bone gap < 20 dB, an intact graft is recognized as an anatomical success [1]. There are a limited number of studies in the literature, reporting the outcomes of endoscopic tympanoplasties. There are no studies in the literature, comparing the functional and anatomical successes of the use of fascial and perichondrial grafts in endoscopic type 1 tympanoplasties. This present study has compared the anatomical and functional outcomes of grafting with the fascia of the temporalis muscle and with the perichondrium of the tragal cartilage in patients undergoing primary transcanal type 1 tympanoplasty with endoscopy.

## Methods

This retrospective study was conducted between January 2015 and July 2017 at our hospital’s ear nose and throat surgery clinic, including a total of 151 patients (80 females and 71 males with a mean age of 26.0 ± 9.3 years in the age range between 18-57) with middle ear risk index (MERI) scores ranging from 1 to 3 and who underwent a transcanal endoscopic type 1 tympanoplasty without tympanomeatal flap elevation. The study was approved by the Ethics Committee of the respective hospital (Ethics Committee no:16/04/2018-E.15902/04). The study was conducted in compliance with the principles of the Helsinki Declaration and Good Clinical Practice Guideline.

The patients were included in the study if they were older than 18 years old and if they had been followed-up at our clinic for at least a year. The patients were excluded if they underwent a mastoidectomy or a tympanoplasty other than the type 1; if they had the following disorders including ossicular chain defects, cholesteatoma or tympanosclerosis; or if they did not appear at the follow-up visits regularly.

All patients had been followed-up at our clinic for at least 12 months. All patients underwent preoperative computed tomography (CT) imaging and audiologic investigations. Temporal CT was performed all the patients to evaluate the status of middle ear pathologies and the neurovascular (high jugular bulb, fallopian canal anomalies, etc.) anomalies. The middle ear pathologies were evaluated endoscopically from tympanic membrane perforation before the operation. All patients were operated under general anesthesia and within the framework of well-established principles of ear surgery.

The following data from the patients were documented including the age, gender, the operated ear, the size of the perforation in the tympanic membrane, the type of graft used, the types and status of middle ear pathologies, the pre-operative and postoperative audiologic test results, the condition of the graft as observed during the postoperative follow-up visits, and the duration of the follow-ups. These data were collected by reviewing the patient charts saved in the hospital records. The status of the middle ear had been previously evaluated in the patients using the MERI developed by Becvarovski and Kartush [16] in the preoperative term. We aimed to standardize the patient data by using the MERI score with the purpose of preventing any inter-group biases. The patients were excluded from the study if they had a MERI score over 3. The tympanic membrane perforations were classified as total (100%), subtotal (>%50), and smaller than 50% (medium).

The patients included in the study were assigned to two groups according to the type of the graft used. The patients were assigned to either the tragal cartilage perichondrium group (Group A) or the fascia of the temporal muscle (Group B). The types of the grafts used in the tympanoplasties were selected upon the experience or preferences of the surgeons. All operations were individually performed at the clinic by two surgeons in compliance with the otologic surgery principles. Before grafting, the perforation in the tympanic membrane was examined by endoscopy in all patients included in the study. A 4 mm 18 cm rigid endoscope (Karl Storz HOPKINS II®) and Karl Storz 24 INCH Full HD® monitor were used in all patients. After reviving the perforation edges, the malleus was de-epithelialized and the grafts were taken. The grafts were created by taking the anterior and posterior perichondria of the tragal cartilage separately, preserving the cartilage itself. All grafts were left to dry before being placed in the tympanic membrane. After the collected grafts were re-shaped so that they would match with the perforation in the tympanic membrane, the grafts were placed over the malleus and under the annulus with an over-underlay technique while observing the ossicular chain. In Group B, the grafts were taken from the fascia of the temporalis muscle by a 2-3-cm supraaural incision. Temporalis muscle fascia grafts were left to dry before being placed in the tympanic membrane as perichondrium. These grafts were then re-shaped depending on the perforation in the tympanic membrane and, while observing the ossicular chain, they were placed over the malleus and under the annulus with an over-underlay technique so that they would close the perforation. The supraaural incision was then sutured in accordance with the anatomical plan. The grafts were supported medially and laterally by gelfoams.

All patients have been routinely given a course of oral antibiotics during postoperative 1 week (selection of type of antibiotics depends on patients sensivity) and a prescription for eardrops untill the gelfoam aspiration from the external ear canal (two times a day). In the first postoperative week, the sutures of the fascias were taken. In the third week postoperatively, the unresolved and persistent particles of the gelfoam in the external auditory canal were aspirated in all patients to allow for examining the tympanic membrane clearly. The postoperative status of the tympanic membrane in the first, third, and 12th weeks was recorded along with the pure audiologic audiometry test results in the postoperative 12th month. The groups were compared according to the pre- and postoperative air-bone gaps and to the status of the tympanic membrane. The success criteria were defined to have an intact tympanic membrane without any retractions or lateralizations and to obtain a reduction in the air-bone gap (ABG) value below 20 dB. ABG value was calculated by averaging the four frequencies (0. 5, 1, 2. and 4 kHz).

### Statistical analysis

In the statistical analyses, NCSS (NumberCruncher Statistical System) 2007 (Kaysville, Utah, USA) program was used. The data were analyzed using descriptive statistics (mean, standard deviation, median, frequency, ratios, minimum, maximum). The comparison of the normally distributed quantitative data between two groups was made using the Student t-test. If the variables did not conform to a normal distribution, Mann Whitney U test was used. The paired samples t-test was used to evaluate the postoperative data in comparison to data collected pre-operatively. Pearson Chi-square test and Fisher Freeman Halton test were used to compare the qualitative data. The level of significance was assessed at a level of *p* < 0.05.

## Results

Considering all study patients, tympanoplasties were performed on the right in 48.3% (*n* = 73) patients and on the left in 51.7% (*n* = 78) patients. Group A consisted of 79 patients while there were 72 patients in Group B. The duration of follow-up ranged from 12 months to 33 months with a mean follow-up period of 16.0 ± 5.2 months. The duration of operation was 48.62 ± 6.88 min in Group A, and it was 46.12 ± 7.64 min in Group B. There were no statistically significant differences in the distribution of the age, gender, localization, MERI scores, the duration of the operation, and the size of the perforation (all *p* values> 0.05) (Table 1).

**Table 1:** Comparison of subject data in the perichondrium and fascia groups

Variables	Grup A (Perichondrium)(*n* = 79)	Grup B (Fascia)(*n* = 72)	*p*
Age (years)	26.81 ± 10.54	23.70 ± 7.74	^*a*^*0.115*
Gender			
-Females	38 (48.1%)	42 (58.3%)	^*c*^*0.208*
-males	41 (51.9%)	30 (41.7%)	
The side operated			
-Right	42 (53.2%)	31 (43.1%)	^*c*^*0.214*
-Left	37 (46.8%)	41 (56.9%)	
MERI score	2.10 ± 1.44	2.32 ± 1.24	^*b*^*0.313*
Graft intact			
Perfore	4 (5.1%)	2 (2.8%)	^*c*^*0.743*
Intact	75 (94.9%)	70 (97.2%)	
Perforation size			
-Medium (25-50)	39 (49.4%)	30 (41.7%)	^*a*^0.284
- Total (%100)	3 (3.8%)	2 (2.8%)	^*a*^0.478
- Subtotal (> 50)	37 (46.8%)	40 (55. 5%)	^*a*^0.142
Mean Operating time (minute)	48.62 ± 6.88	46.12 ± 7.64	^*d*^0. 318
Mean follow-up time (month)	16.44± 5.43	17.38± 5.01	^*d*^0.307

^a^Independent Samples Test

^b^Yates Continuity Correction

^c^Fisher’s exact test

^d^ Mann-Whitney U-test

**Fig. 1:**
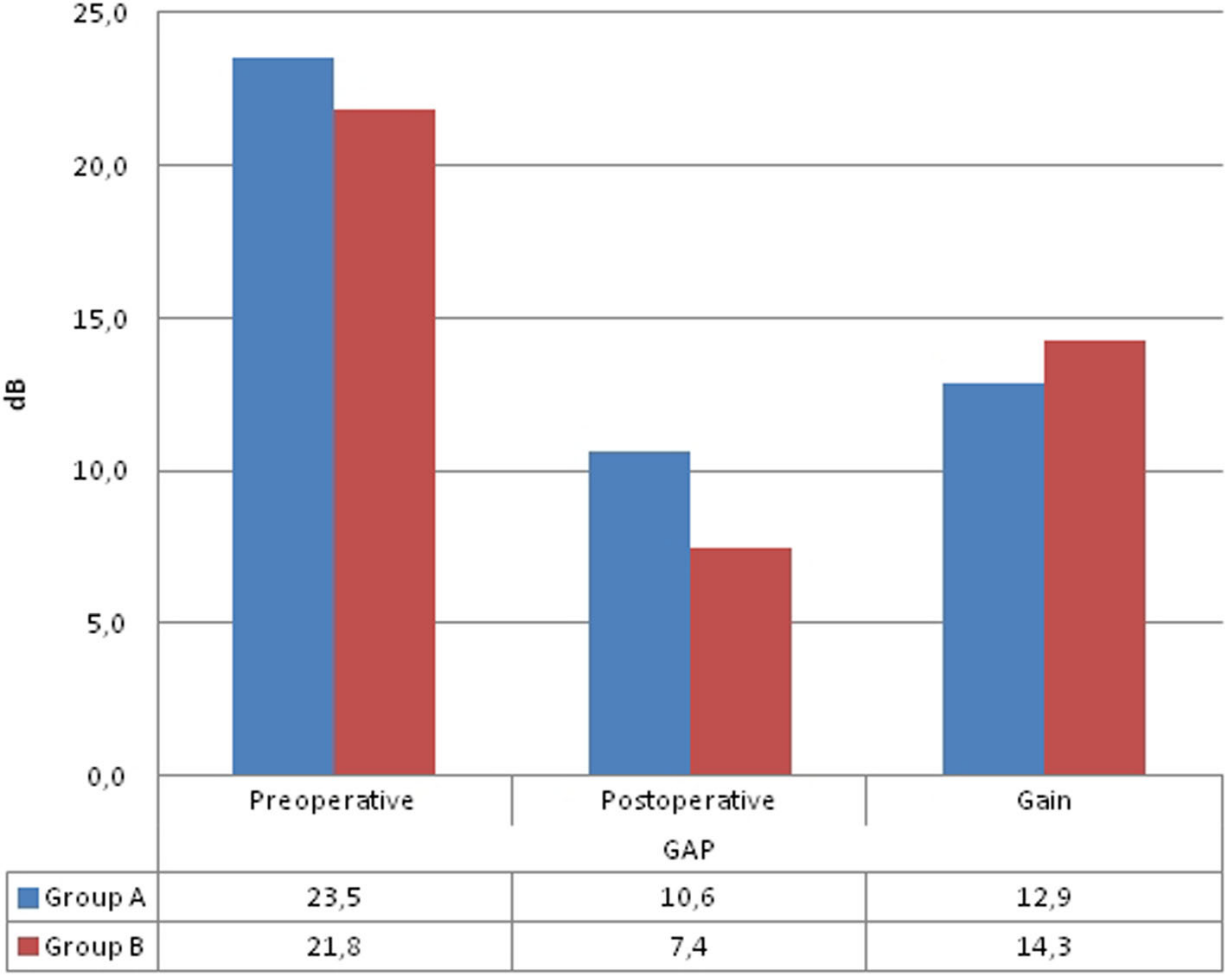
Comparison of air-bone gap between the perichondrium and fascia groups

The mean preoperative and postoperative air-bone gap values were 23 [5]. ± 6.9 dB and 10.60 ± 4 [5]. dB in Group A, respectively. The mean preoperative and postoperative air-bone gap values were 21.8 ± 7.3 dB and 7.4 ± 6.1 dB in Group B, respectively (Table 2). The pre-operative air-bone gap values of Group A and B did not show a statistically significant difference (*p* = 0.073). The value of postoperative air-bone gap in Group B was significantly lower than that of Group A (*p* = 0.0001). The postoperative air-bone gap value showed a significant reduction in Group A compared to its respective value in the pre-operative period (*p* = 0.0001). The postoperative air-bone gap value showed a significant reduction in Group B compared to its respective value in the pre-operative period (*p* = 0.0001). The postoperative improvement in the air-bone gap value did not demonstrate a significant difference between Group A and B (*p* = 0.202) (Fig. 1).

**Table 2:** Comparison of air-bone gap and the hearing gains between the two groups pre- and postoperatively

Air Bone Gap	Preoperative ABG (dB)	Postoperative (dB)	P^a^	Gain(median)	p^b^
Group; mean ± SD					
Group A (N:79)	23.54 ± 6.89	10.59 ± 4.47	0.001**	12.88 ± 6.95	0.202
Group B (N:72)	21.83 ± 7.32	7.44 ± 6.11	0.001**	14s.28 ± 8.41 s	0.073

^a^Paired Samples test

^b^Mann Whitney U test

**p < 0.01

*p < 0.05

^a^; Comparison ABG between two groups pre- and postoperatively

^b^: Comparison between two groups in terms of gain

The graft retention rates without any retractions or lateralizations according to the success criteria were 94.9 and 97.2% in Group A and in Group B respectively. There were no statistically significant differences between the two groups in terms of the graft retention success rates (*p* = 0.743) (Table 3). Considering the perforation size, there were no statistically significant differences between the two groups in terms of the graft retention success rates (all *p* values > 0.05) (Table 4).

**Table 3:** Comparison of success rates between the groups

	Group A(N:79)	Group B(N:72)	*P*
Graft success	94.9% (N:75)	97,2%(N:70)	^*a*^*0.743*
Hearing success (ABG ≤ 20 dB)	87.34%(N:69)	93,05%(N:67)	^*a*^*0.942*

^a^Fisher’s exact test

**p < 0,01

**Table 4:** Greft retention success of each groups according to perforation size

Perforation size	Group A (n:79)	Group B (n:72)	^*a*^*p*
-Medium (25-50%)	100%	96.6%	*0.224*
- Subtotal (> 50%)	91.8%	100%	*0.096*
- Total (100%)	66.7%	50%	*0.132*

^a^Independent Samples Test

## Discussion

With the continuing developments introduced into the surgical practice, tympanoplasty is one of the most frequently applied otologic surgery procedures at otorhinolaryngology clinics [1, 3]. Otologists do research in order to improve the success rates of tympanoplasties and to reduce the frequencies of revision surgeries. The advances of technological developments in the medical field allowed for operations to last shorter and they allowed for improvements in the success rates of the operations and in the quality of lives of the patients in the postoperative period.

Endoscopic ear surgery was applied first in an excision of a cholesteatoma and in a myringoplasty procedure. Then, the endoscope has been used in surgeries involving the middle ear, in ossiculoplasties, tympanoplasties, and in cochlear implantation operations [17]. The available studies demonstrate that the endoscopic ear surgery is a reliable method with minimal complication and morbidity rates. The use of the endoscopic techniques in the middle ear operations has led to the development of the concept of minimally invasive surgery. This enabled the surgeons to avoid mastoidectomies, retroauricular incisions, and tissue dissections in selected cases [18–20].

Furthermore, the microscopic ear surgery method presents with significant advantages. It facilitates mastering the surgical field by allowing a binocular vision. It is one of the major advantages that a surgeon may use her or his both hands during the surgery. However, it is more challenging to evaluate the deeply located anatomical structures including the facial recess, the tympanic sinus, the attic, and the epitympanic area by microscopy. In addition, the use of a microscope requires an increased number of dissections to enhance the field of view, sometimes necessitating a detouring of the posterior wall of the external ear canal [18, 19].

On the other hand, the use of an endoscope facilitates the visual quality in the surgical field and the use of diverse degrees of endoscopes allows for an easier examination of the deeper anatomical structures without the need for dissections or detouring of the posterior wall of the external ear canal. The advantages of endoscopic tympanoplasty can be listed in the following that, when the endoscope is used, it is not necessary to manipulate the head of the patient, it is easier to evaluate the deep anatomic structures from different angles, the operation time is shorter, the postoperative pain is at a lesser intensity, and the hospitalization time is shorter. However, there are also disadvantages of the endoscopic ear surgery. The direct or thermal effect of the endoscope light may cause damage to the external auditory canal and middle ear structures. Also using one of the hands only during the endoscopic tympanoplasty procedure may hamper surgical manipulations. In the case of a sudden hemorrhage, the image provided by the endoscope can be disrupted [21, 22]. However, in the endoscopic technique, the limitations brought by only using one hand will be reduced with increasing experience. Moreover, the current information in the literature demonstrates that endoscopic tympanoplasties can be performed successfully in the hands of experienced surgeons [23].

There are several studies in the literature comparing the anatomical and functional outcomes of the microscopic and endoscopic tympanoplasty techniques. Jiyothi et al. [22] compared endoscopic and microscopic myringoplasties in a study, reporting the anatomical success rate as 91.67% for endoscopic tympanoplasty and as 93.3% for microscopic tympanoplasty. The functional success rates were reported to be 91.67 and 93.3% for endoscopic and microscopic tympanoplasties, respectively, in the same study. The studies report that endoscopic tympanoplasty can be an alternative surgical method to microscopic tympanoplasties. However, there are few studies in the literature, comparing the use of different grafts in endoscopic tympanoplasty procedures. In the comparative study of Choshan et al. [24] reported that cartilage graft has an excellent anatomical result, perfect stability and good functional outcome in endoscopic tympanoplasties. However, the grounds of insufficient information we have today results from the fact that the endoscopic tympanoplasty is a method, which has just been introduced for use with its requirements for experience. This present study has compared the anatomical and functional (audiologic) outcomes of grafting with the fascia of the temporalis muscle and with the perichondrium of the tragal cartilage use in endoscopic tympanoplasty, which is relatively a new technique. The anatomical and functional success rates were 94.9 and 87.34%, respectively, in the perichondrium group. On the other hand, in the fascia group, these rates were 97.2 and 93.05%, respectively. There was not a statistically significant difference between the two groups in terms of the anatomical or functional success rates. With the use of both types of grafts, significantly successful outcomes were achieved both anatomically and functionally. These rates are consistent with the results of the studies using a microscopic tympanoplasty technique. In a study comparing the anatomical and functional results of fascia and perichondrium used in microscopic type 1 tympanoplasty, Dabholkar et al. [25], reported the anatomical success rates as 84 and 80% for the fascia and the perichondrium, respectively. In that study, the authors defined the functional success criteria as a reduction in the value of the air-bone gap below 10 dB and reported the functional success rates as 76.2 and 76% for the fascia and perichondrium, respectively.

A number of factors are involved in tympanoplasties, resulting in diverse outcomes in the studies comparing the success rates of the grafts. These factors may include the number of patients included in the study, the surgical technique employed, the age of the patient, the size of the perforation in the tympanic membrane, the simultaneous interventions of diverse types applied in the middle ear, co-morbid middle ear pathologies, and the duration of follow-up periods of the patients [1, 6, 8]. Adult patients were included in this present study. Although the number of patients included in this study is relatively lower, the longer follow-up periods increase the reliability of the study. The anatomical and functional success rates gradually decline in tympanoplasties. All patients had been followed-up for at least 12 months in this present study, with the evaluation of their postoperative 12th-month outcomes. Considering perforation size, both grafts have been similar graft retention success rates. Both grafts seem to be proper for the every perforation in endoscopic tympanoplasties.

Although the study has provided very valuable data, it has some limitations as well. The major limitations are the retrospective design of the study and the small number of patients included. In addition, having two individual surgeons in one study is considered as a limitation. However, the experience of more than 5 years of both of these surgeons in otologic surgeries and their performing the operations within the frame of the principles of otologic surgery relatively reduce the negative potential of having two surgeons leading to untoward effects which may influence the surgical outcomes. However, tympanoplasties in each group were performed by one surgeon and the success of tympanoplasties did not differ between the two groups. There is a need for further studies conducted with larger study populations. Further studies using diverse types of grafts and comparing the outcomes of endoscopic tympanoplasties will help reduce the gap of knowledge.

## Conclusion

In this present study, the anatomic and functional success rates of different grafts were compared in the patients undergoing transcanal endoscopic type 1 tympanoplasty. Perichondrium and fascia were found out to be accepted for use in endoscopic tympanoplasties. There is a need for further studies comparing different types of grafts in endoscopic tympanoplasties.

## Data Availability

The data and materials of this study are available from the corresponding author for a reasonable request.
